# The launch of *Journal of Extracellular Vesicles* (JEV), the official journal of the International Society for Extracellular Vesicles – about microvesicles, exosomes, ectosomes and other extracellular vesicles

**DOI:** 10.3402/jev.v1i0.18514

**Published:** 2012-04-16

**Authors:** Jan Lötvall, Lawrence Rajendran, Yong-Song Gho, Clotilde Thery, Marca Wauben, Graca Raposo, Margareta Sjöstrand, Douglas Taylor, Esbjörn Telemo, Xandra O. Breakefield

In 2011, researchers around the world interested in extracellular vesicles (EV) joined forces and founded the International Society for Extracellular Vesicles (ISEV). Membership has grown to approximately 750 in eight months, and the Society's first meeting will take place in Gothenburg, Sweden, on 18–21 April 2012. Already approximately 500 participants have been attracted to this event. These are signs of rapid expansion in global research in the field of EV.

It is with great pleasure that the interim board of ISEV launches the Society's official journal: *Journal of Extracellular Vesicles* (JEV). Scientific publications in the field of extracellular research have been dispersed across many journals, including in some publications lacking the necessary expertise to judge methodologies and nomenclature in the field. Furthermore, following a survey of the new membership and outreach to the community, the need for a journal in this field became evident. In this climate it was an easy decision for the interim board of ISEV to launch the journal, and also that it should be published according to the open access principles.

*Journal of Extracellular Vesicles* aims to publish high-impact research relevant to scientists involved and interested in EV in the form of original research articles as well as review articles. Manuscripts selected for publication should contain data of high quality that advance the field and contribute to an in-depth understanding of the processes under study. Areas of interest include, but are not limited to:

- technological advances in isolation, quantification and characterization of EV, including exosomes, microvesicles, ectosomes, apoptotic bodies and any other type of EV;
- biological functions of EV in health and disease;
- intracellular molecular mechanisms of EV production and release, including membrane trafficking and protein sorting;
- EV as biomarkers;
- signal transduction, uptake and propagation of EV;
- stem cell biology and EV;
- informational databases as EV resources, including large sets of data on genomics, lipidomics, proteomics, high-throughput screening data and other techniques.

The scope of *Journal of Extracellular Vesicles’* is well reflected in the first scientific publications in the journal, which include work on both what are evidently exosomes as well as larger microvesicles. Interestingly, Bobrie and colleagues show that vesicles isolated via “a classical exosome isolation procedure” actually contain at least two separate types of extracellular nano-vesicles that appear to be of different intracellular origin (1). This is further supported by a study by van der Vlist and colleagues (2), showing that CD4 T-lymphocytes can produce distinct populations of nanosized vesicles. Furthermore, Aliotta and colleagues (3) show that the lungs and bone marrow can communicate via EV in a way that influences long-term bone marrow cell phenotype. Ekström and colleagues (4) suggest that one possible mechanism could be the transfer of RNA from mast cells to bone marrow CD34 cells, including both delivery of mRNA and microRNA. Interestingly, the protein and RNA profile of cell-derived vesicles change in different situations of stress, which has the potential to fundamentally influence the biological activity of the vesicles, as suggested by de Jong and colleagues (5). The aim of JEV to be a platform for communicating informational databases is exemplified in the article by Simpson and colleagues (6), discussing the ExoCarta database as a resource for exosome research; they propose to further develop this repository toward what they have named Vesiclepedia, including proteomics and lipidomics of all types of EV.

We expect that JEV will become the key forum for exchanging research in the field. JEV is launching strongly, with six publications and a supplement consisting of 265 abstracts for the International Society for Extracellular Vesicles 2012 Meeting (7). In light of the rapid flow of submissions we are already experiencing, JEV looks to be on the fast-track towards being included in PubMed Central/PubMed. The journal and the Society are here to serve the broad community of researchers working on different aspects of EV according to different models and diseases. We look forward to your contributions.
